# Acellular bioactivity and drug delivery of new strontium doped bioactive glasses prepared through a hydrothermal process

**DOI:** 10.1039/d2ra02416k

**Published:** 2022-05-19

**Authors:** Salwa El Baakili, Khalil El Mabrouk, Meriame Bricha

**Affiliations:** Euromed Research Center, Euromed Engineering Faculty, Euromed University of Fes, Eco-Campus Fes-Meknes Road 30030 Fes Morocco k.elmabrouk@ueuromed.org

## Abstract

This work aims to study the kinetics of apatite layer formation on the surface of strontium doped binary bioactive glasses (BG: 63S37C) prepared for the first time by a hydrothermal process and evaluate their potential for drug loading and release using ibuprofen (IBU) as an anti-inflammatory drug vector. First, the binary glass 63S37C was doped with various amounts of strontium, from 0.2 to 1 mol%. Subsequently, the amorphous state of the samples and the microstructure were assessed by TGA, XRD, FTIR, ICP-AES, and SEM-EDS. Next, the *in vitro* bioactivity was evaluated by following the surface morphology and composition changes of soaked samples for up to 14 days at 37 °C in simulated bodily fluid (SBF). Finally, SEM-EDS spectroscopy showed clearly the appearance of needle-shaped apatite on amorphous glass substrates at the earlier stages of immersion for bioglasses doped with strontium. These findings are also confirmed with XRD and FTIR analysis. Furthermore, 63S37C BG proved that the drug release increased with increasing strontium content. Altogether, this novel class of bioactive glasses may be considered to have a promising future for biomedical applications.

## Abstract 2

This work aims to study the kinetics of apatite layer formation on the surface of strontium doped binary bioactive glasses (BG: 63S37C) prepared by a hydrothermal process and evaluate their potential for drug loading and release.

## Graphical abstract

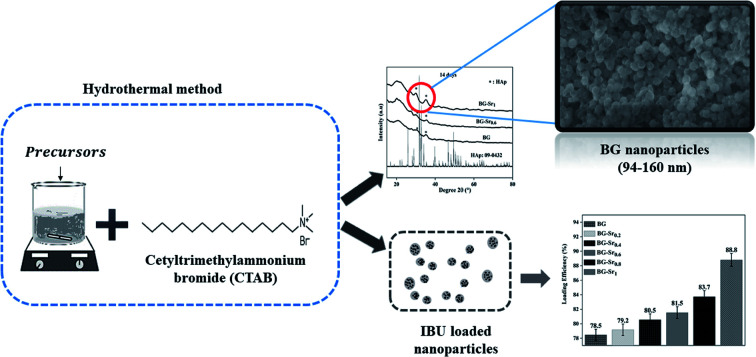

## 1. Introduction

The last 40 years have seen real progress in bone disease medicine and surgery. The application of bioceramic materials and bioglasses has contributed to this success. As a result, they have attracted tremendous attention since their invention by L. Hench *et al.* in 1969. This led to a revolution in developing new bioglass compositions (BG) for hard tissue engineering applications. Glass development is classified into four phases: discovery to innovation through clinical application and tissue regeneration.^1,2^ Bioglasses are amorphous materials, presenting many promising properties, such as biodegradability, biocompatibility, and osteogenesis. Moreover, BG exhibit angiogenic and antimicrobial properties due to the metallic ions released upon dissolution and activated specific biological responses.^3,4^

Glass bioactivity is a phenomenon of two primary stages and differs from the surface to the bulk. In the first one, some specific reactions on the surface occur spontaneously, in contact with simulated body fluid. In the second stage, a new apatite layer is formed, responsible for biocompatibility and bio-conductivity reactions, in hard and soft tissues, within only the first 10 to 30 days after immersion.^5^ In this sense, the bioactivity design of bioglass depends on the chemical composition and structural and textural properties.^6^ The precursor's nature highly manages these properties, the synthesis methods, and the morphology of the synthesized bioglasses.^7,8^ For example, the final shape and size of particles depend on the chemical method's factors, such as pH value, ammonia solution concentration, and reagents ratio, which implicitly influence the process of hydrolysis and condensation.^9^ Likewise, aqueous ammonia solution in the sol–gel process with different concentrations (1 mol l^−1^ and 3 mol l^−1^) results in various morphology of the final product (nanosphere and radial). This demonstrates that the particle rapport ratio “length-diameter ratio” increases with the aqueous ammonia concentration. In addition, the fast BG gel production rate results in larger nanoparticles.^3,8^

Meanwhile, V. Regi and Martinez studied the relationship between bioactivity and chemical composition using the sol–gel method.^10^ They found that hydroxyapatite (HAp) layer deposition on the glass surface is influenced by the SiO_2_ (50–90 mol%) content of the glass composition. The glasses with lower SiO_2_ (50–70 mol%) and highest calcium content present a very high rate of HAp formation.^10^

Till 1992, the glass was usually produced by melting and only focused on 45S5 bioactive glasses. However, the melting process presents many drawbacks, such as high-temperature processing (typically around 1500 °C), the evaporation of volatile components,^4,11^ the high processing costs of oxides and additives, and limited compositional range.^12,13^ The first synthesis of bioactive glass 45S5 realized by L. Hench explained the restricted compositional range of bioactive glasses produced by quench melting, with slower bonding rates ranging from 52 to 60 wt% of SiO_2_. Bioglasses containing more than 60% SiO_2_ do not bind and are bio-inert.^14^ However, these compositions may be extended to more significant percentages of SiO_2_ (up to 90 mol%) by a sol–gel method, promoting *in vitro* and *in vivo* potential.^15,16^ Researchers were recently oriented to use the sol–gel method to synthesize glasses as an alternative to the melting route.^17^ Tabia *et al.* adapted the sol–gel method to produce magnesium doped nanoparticles with high surface area (311 m^2^ g^−1^) and high bioactivity (after only 3 hours). Their bioglasses are served as drug delivery for amoxicillin.^18^

The sol–gel method offers good features at a lower processing temperature, such as high surface area and controlled porosity.^19,20^ Li *et al.*^16^ examined the SiO_2_ limit beyond which the powders lost their bioactivity. They found that the sol–gel technique promotes bioactivity even for a higher SiO_2_ (≥90 mol%) compared to traditional melting methods.^16^ Moreover, the first bioactive glass with highly order mesostructured was synthesized, in 2004, by adding a block copolymer and surfactant during the sol–gel process.^19^ Likewise, Hu *et al.* used cetyltrimethylammonium bromide “CTAB” as a templating agent in the sol–gel process to produce hollow bioactive glass sub-micron spheres, with a high specific surface area, of about 684.608 m^2^ g^−1^ and relatively homogeneous size.^20^ However, traditional bioactive glasses synthesis could only control mesoporous sizes and morphologies in a limited range, restricting their application scope for delivering therapeutic molecules.^21^ As a result, developing a unique and straightforward approach to getting a bioactive glass with a wide range of adjustable mesostructures remains a big challenge.

Only a few studies tried to prepare bioactive materials using hydrothermal synthesis. It is defined as a heterogeneous solution-reaction-based, in the presence of a solvent (aqueous or non-aqueous), at a high temperature (100 °C) and a wide pressure range, higher than 1 atmosphere, in a closed system.^12^ In particular, hydrothermal synthesis can produce nanomaterials unstable in high temperatures and high vapor pressures with minimal material loss. In addition, through a liquid phase or multiphase chemical process, this method offers an opportunity to control the compositions and morphologies of the produced nanomaterials carefully. This special issue has published several research findings on the hydrothermal synthesis of nanoparticles, nanorods, nanotubes, hollow nanospheres, and graphene nanosheets.^22^ However, only three of them are concerned the bioactive glasses.

In the same way, a green synthesis by the hydrothermal process was recently used for the first time by Hoa *et al.*^23^ to produce nano-spherical particles. As a result, visible aggregations of almost spherical particles with diameters ranging from 20 to 30 nm were observed, in addition to exciting bioactivity when immersed in simulated body fluid (SBF) for seven days. Similarly, Ta Anh Tuan *et al.* produced a binary bioactive glass (70SiO_2_–30CaO) using the acid-free hydrothermal process with typical mesoporous structures consisting of aggregates nanoparticles.^24^ Likewise, Qiming Liang *et al.*^8^ studied the effect of the synthesis method parameters on the particle's morphology and size. They found that BG with various morphologies could be controlled by changing the aqueous ammonia concentrations and the synthesis conditions (pressure and temperature). As a result, two types of novel BG particles were obtained. One displayed spherical morphology with radial mesostructured, and others exhibited pineal morphology with lamellar mesostructured.

Thanks to the specific morphology and structure of bioactive glasses, research and developments extended the use of BG to investigate their ability as delivery supports for therapeutic molecules (drugs, proteins…) in biomedical applications. According to the literature reviews, it is known that the released ions from bioglass contributed to a highly active apatite layer, up-regulation of osteogenic gene expression, and stimulated osteoblast proliferation and differentiation.^25,26^ Incorporating different ions, such as magnesium,^27,28^ copper,^29,30^ or silver,^31^ into the bioglass composition is the best way to improve physio-chemical, mechanical, bacterial, and biochemical properties. Metallic ions such as strontium have emerged as potential therapeutic agents because of their inhibitor behavior towards osteoclast activity and osteoporosis.^32^ Moreover, the strontium ion is preferable for increasing the material's biological functionality,^33^ osteogenesis and angiogenesis activity.^34,35^ Therefore, strontium incorporation in bioactive glasses may be an effective way to deliver a steady supply of strontium ions to a bone defect site for osteoporotic patients.^33^ Notably, Sr^2+^ ions have shown a remarkable effect on glass properties during its substitution. For instance, Fredholm *et al.* studied the influence of strontium on calcium substitution (0–100 mol%) in a bioactive glass. The results demonstrate that the glass network was expanded, the apatite formation was enhanced significantly in the fully Sr-substituted glass, and the proliferation and the osteoblasts control.^36^ Other studies have also been carried out on strontium-containing sol–gel glasses. They indicate that the dissolution rate decrease with strontium content in binary glasses.^37^

**Fig. 1:**
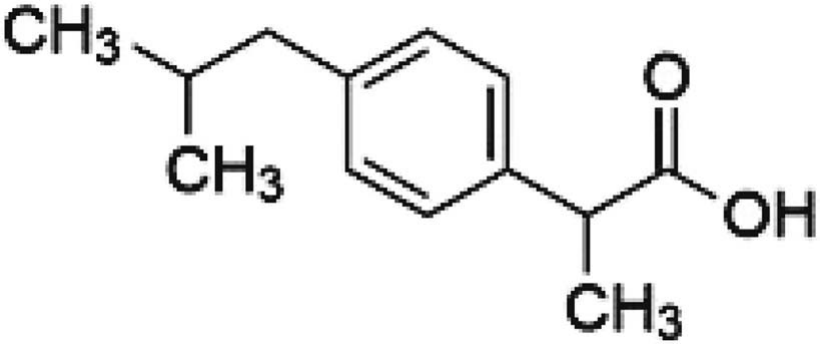
Chemical structure of ibuprofen “IBU”.

During the past decade, much effort has gone into developing novel drug loading/release systems in BG, which control the release time and predict therapeutic response to improve bioactive glass properties. An efficient delivery system should transport the desired molecule drugs to the targeted cells or tissues, releasing the drug in a controlled manner.^38^ This work aims to explore the potential property of the bioactive glass 63S37C with different amounts of Sr dopants to act as convenient storage for controlled drug delivery systems. Ibuprofen (IBU) (Fig. 1) molecule was chosen as a drug model for the loading and release study of antibiotics due to its pharmacological activity and molecular size (1.0 nm × 0.5 nm). In addition, IBU is also well-known as a nonsteroidal anti-inflammatory drug (NSAID) with an analgesic property.^12,38^

According to the above, this study aims to evaluate the incorporation of strontium with several concentrations (0.2 to 1 mol%) in a new binary bioactive glass (BG: 63S37C) using the hydrothermal process. In addition, the potential of the nanoparticles to form bioactive layers on their surface was also evaluated, and their potential for drug loading and release using ibuprofen (IBU) was also studied.

## 2. Experimental section

### 2.1. Materials

Tetraethyl orthosilicate Si(OCH_2_CH_3_)_4_ (TEOS ≥ 99%, VWR), calcium nitrate tetrahydrate (Ca(NO_3_)_2_ ≥ 99% 4H_2_O, SOLVACHIM), strontium nitrate (Sr(NO_3_)_2_, 99%, Alfa Aesar), cetyltrimethylammonium bromide (C_19_H_42_BrN, CTAB), ethanol (C_2_H_6_O, 99%) and ammonia hydroxide (NH_4_OH) were used to produce the bioactive glass powders. For simulated body fluid (SBF) and phosphate-buffered saline (PBS) preparation, the following chemicals were used: calcium chloride (CaCl_2_), potassium chloride (KCl), sodium chloride (NaCl), sodium sulfate (Na_2_SO_4_), sodium bicarbonate (NaHCO_3_), sodium phosphate dibasic (Na_2_HPO_4_), magnesium chloride hexahydrate (Mg(Cl)_2_, 6H_2_O), potassium phosphate dibasic trihydrate (HK_2_PO_4_, 3H_2_O), trizma (C_4_H_11_NO_3_) and hydrochloric acid (HCl). All the chemical precursors listed were used without further purification.

### 2.2. Synthesis of bioactive glass 63SiO_2_–(37 − *x*)CaO–*x*SrO

**Fig. 2:**
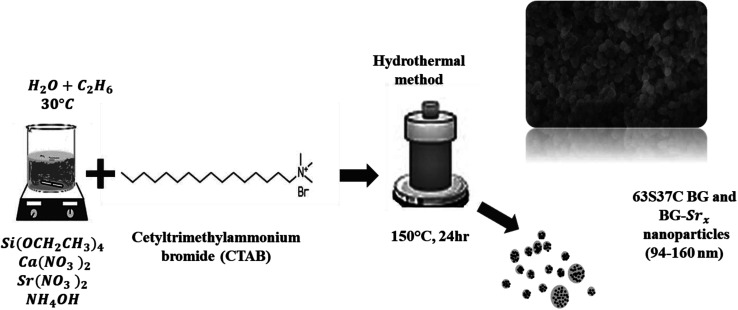
Schematic of the 63S37C BG and BG-Sr_*x*_ nanoparticles produced by hydrothermal process.

The hydrothermal method was used to successfully synthesize a new composition of strontium-substituted bioglass at 30 °C, using CTAB as a structure-directing agent (Fig. 2).

The synthesis was performed as described previously^18,23^ with slight modifications. Briefly, the surfactant was dissolved in 120 ml of distilled water and ethanol, and then ammonium hydroxide was added as a catalyst. Afterwards, proper amounts of (Ca(NO_3_)_2_, 4H_2_O), and strontium nitrate (Sr(NO_3_)_2_) were added to the mixture depending on the glass composition, as mentioned in Table 1, followed by continuous stirring for 30 minutes. Then, TEOS was added drop by drop, and the resultant mixture was allowed to react for an additional 30 min. The precipitate was then put in a Teflon-lined stainless autoclave. The system was heated at 150 °C for 24 hours. The resulting product was filtered and then dried for 1 day at 80 °C. Finally, the heated sample was sintered at 700 °C for 6 hours using a heating rate of 1 °C min^−1^ to remove the remaining surfactant molecules and solvents from the samples.

**Table 1:** Chemical composition and sample nomination

Bioglass code	BG	BG-Sr_0.2_	BG-Sr_0.4_	BG-Sr_0.6_	BG-Sr_0.8_	BG-Sr_1_
SiO_2_ (mol%)	63	63	63	63	63	63
CaO (mol%)	37	36.8	36.6	36.4	36.2	36
SrO (mol%)	0	0.2	0.4	0.6	0.8	1

### 2.3. Physicochemical characterization

Structural characterization of synthetic bioactive glass was analyzed using X-ray diffraction (XRD) spectroscopy, with an automated X-ray powder diffractometer (Panalytical) using CuKα radiation at a voltage and current of 45 kV and 40 mA, respectively.

Fourier transformed infrared spectroscopy in Attenuated Total Reflection mode (FTIR-ATR) “IS50 spectrometer” to identify the functional groups. The samples were analyzed in the range of 400 to 4000 cm^−1^ with a resolution of 4 cm^−1^.

Thermogravimetric analysis (TGA) was realized to predict the thermal behavior of the samples as a function of temperature and obtain the suitable sintering temperature. The dried samples were subjected to thermal degradation tests using the “TA Instruments Q500” thermal analyzer. TGA thermograms were obtained by heating the samples from room temperature to 1000 °C at a heating rate of 10 °C min^−1^ under a constant flow of air atmosphere.

The morphology of bioactive glass was observed by QUATTRO Environmental Scanning Electron Microscope (ESEM-FEG, ThermoFisher Scientific, USA). The images were analyzed with ImageJ software (National Institute of Health, USA).

The elemental analysis of the synthesized samples was analyzed using inductively coupled plasma-atomic emission spectroscopy “ICP-AES; JobinYvon Horiba-Ultima 2 apparatus”. The measurements were repeated three times, and the average is represented in the results section.

### 2.4. Bioactivity in acellular medium

The *in vitro* bioactivity tests were performed by soaking the glass samples in simulated body fluid (SBF) for various periods (3, 7, and 14 days). SBF is a synthetic solution with an ionic concentration similar to human blood plasma (Table 2).

**Table 2:** Ion concentration (mM) in SBF and human blood plasma^27,39^

Ion	Na^+^	K^+^	Mg^2+^	Ca^2+^	Cl^−^	SO_4_^−^	HCO_3_^−^	HPO_4_^2−^
Plasma	142	5	1.5	2.5	103	0.5	27	1
SBF	142	5	1.5	2.5	147.8	0.5	4.2	1

It was synthesized according to Kokubo's method.^18,27,39^ First, the powder samples were soaked in SBF in clean and sterile bottles at 37 °C under continuous stirring at a 2 mg ml^−1^ concentration. Then, after each selected period, the powder samples were filtrated, washed with distilled water, dried at 40 °C for 24 h, and characterized using X-ray diffraction (XRD) spectroscopy, Fourier transformed infrared spectroscopy, and scanning electron microscopy.

### 2.5. pH measurement

The pH variation was measured *via* pH meter (Seven Compact pH/Ion meter – pH METTLER TOLEDO) on different days of immersion. Each measure is repeated three times to calculate the median value, presented in the results and discussions section.

### 2.6. Ibuprofen loading and delivery behavior

#### 2.6.1. Drug loading

The drug loading ability of BG was calculated based on UV-Vis spectroscopy at a wavelength of 264 nm. A series of calibration solutions were prepared, containing 0.005 to 0.05 mg ml^−1^ of IBU in ethanol as a solvent, and their UV absorbance was determined at 264 nm, giving a linear plot (calibration curve) of absorbance against ibuprofen concentration with an *R*^2^ factor of 0.99. The IBU loading of BG was determined by adding 50 mg of BG and BG-Sr_*x*_ to a solution containing 50 mg of ibuprofen in 20 ml of ethanol at room temperature. The mixture was magnetically stirred for 48 hours to ensure the drug equilibration on the carrier. The BG and BG-Sr_*x*_ powders absorbed with IBU were separated from the solution by centrifugation at 10 000 rpm and then washed and dried at 60 °C for 24 hours. The concentration of unloaded IBU was calculated by measuring the absorbance of the supernatant after centrifugation. The composition loading efficiency (*E*) was determined by using the following equation:^18^1
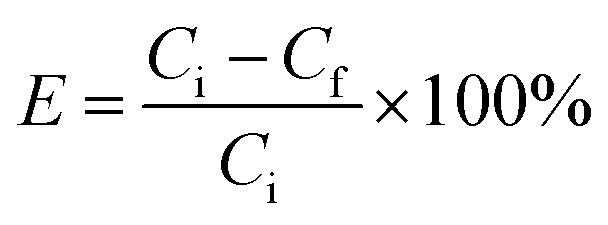
where:- *C*_i_ is the initial concentration of IBU.- *C*_f_ is the final concentration of IBU after drug loading.

#### 2.6.2. Drug release

The ions released from the tested samples into phosphate-buffered saline PBS (pH = 7.4) are measured. The release of IBU from the drug-loaded BG and BG-Sr_*x*_ powders into PBS solution was determined by dispersing 0.1 g of BG powders loaded with IBU into 20 ml of PBS and incubating at 37 °C under stirring. After each selected period, from one hour to 28 days, 4 ml was withdrawn and filtered, and another 4 ml of fresh PBS was added to the solution, keeping the total volume at 20 ml. The concentration of the released drug was determined as a function of soaking time by UV-Vis spectroscopy at 264 nm. The cumulative release profile of IBU was determined according to the following equation:^40^2
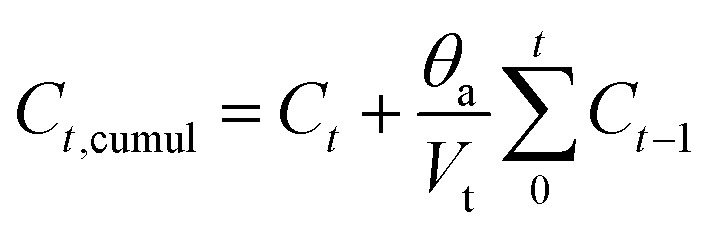
where:- *C*_*t*,cumul_ is the cumulative concentration.- *C*_*t*_ is the concentration at time *t*.- *C*_*t*−1_ is the concentration at time *i* − 1.- *θ*_a_ is the volume of the withdrawn solution;- *V*_t_ is the volume of the total solution.

## 3. Results and discussion

### 3.1. Characterization of bioactive glass

#### 3.1.1. Thermal behavior

**Fig. 3:**
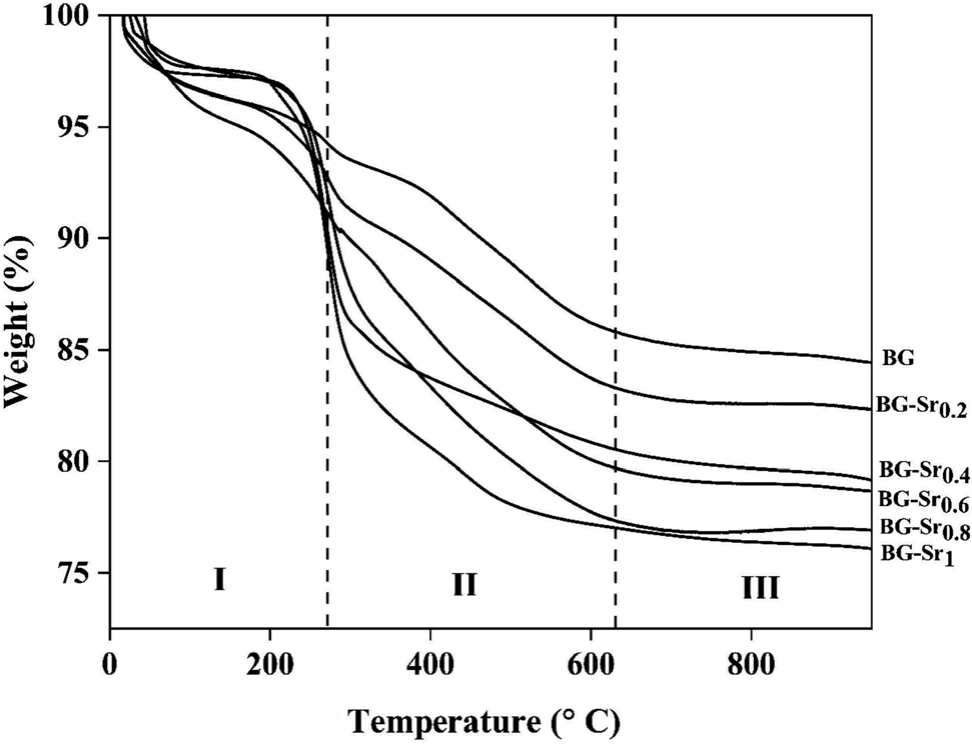
TGA curves of as dried bioactive glass.

The thermogravimetry curves of dried samples are presented in Fig. 3, which shows three mass losses in different temperature ranges. The first range is from 50 to 250 °C, attributed to the loss of physically adsorbed water molecules. The second one is from 250 to 650 °C and is related to eliminating residual CTAB template, residual nitrate, alkoxide groups, and the ethanol residue, which did not react during the hydrothermal synthesis. The weight losses in this area, are mainly confined to the different concentrations of CTAB and nitrate decomposition inside each sample. The third interval, located between 650 to 800 °C, is attributed to the crystallization of bioactive glass. Above 670 °C, no mass loss was observed. Therefore, the suitable temperature for glass sintering is around 700 °C. The obtained result shows that the weight loss increased as the SrO content in the glass increased (Table 3). This demonstrates the effect of SrO addition on the thermal properties of synthetic glasses, as observed in the previous study.^41–46^

**Table 3:** Weight loss of BG doped glass during TGA analysis

Sample acronym	Weight loss (%)			
	I	II	III	Total
BG	5.95	8.36	1.16	15.46
BG-Sr_0.2_	6.67	9.86	1.11	17.69
BG-Sr_0.4_	5.92	13.62	1.31	20.75
BG-Sr_0.6_	7.78	12.80	0.81	21.39
BG-Sr_0.8_	5.39	17.31	0.25	23.13
BG-Sr_1_	6.42	16.67	0.93	24.02

#### 3.1.2. XRD analysis

**Fig. 4:**
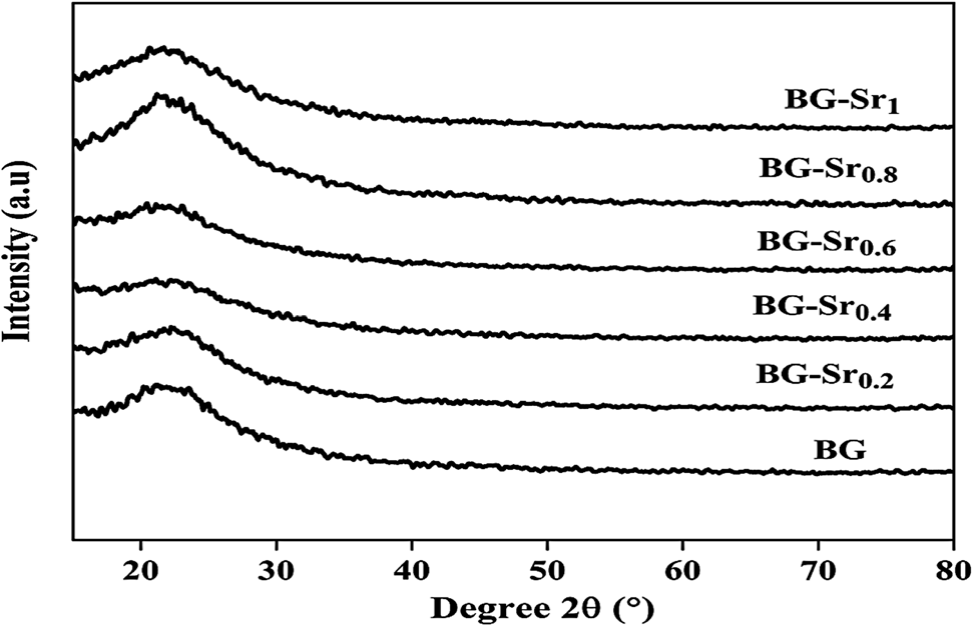
XRD patterns of the glasses after heat treatment at 700 °C.

The XRD patterns of virgin BG and Sr-doped BG are presented in Fig. 4. All compositions synthesized exhibited a broad peak between 20 °C and 30 °C after heat treatment at 700 °C. It describes the absence of any crystalline phase and the presence of an utterly amorphous silicate, characteristic of the amorphous phase.^47^ It is also essential to notice that all the patterns are almost identical but reveal a slight difference in terms of intensity. This suggested that strontium ions were successfully introduced into the glass network. This difference is due to the increased amount of strontium. These results confirm the successful synthesis of the binary 64S36C and the ternary bioactive glasses 63SiO_2_–(37 − *x*)CaO–*x*SrO using the hydrothermal method.

The same results were already obtained by Galliano *et al.*^48^ They studied the effect of Sr incorporation ions in the glass network. They found that alkaline earth metals do not influence the species distribution in the silica network. They suggest that the structure of the silicate arrangement remains unchanged when calcium is partially replaced by strontium.^48^ This agrees with other studies, Massera *et al.*^49,50^ and Taherkhani *et al.*^49,50^ They indicated that Sr incorporation does not critically impact the general glass structure, presented as a network forming and modifying units.^49,50^ This is because the two balanced positive charges create two tetrahedrons linked by ionic bonds. Therefore, glasses improve their chemical durability and network connectivity.^45^ The same findings were raised by Bui Thi Hoa *et al.*^23^ and Ta Anh Tuan *et al.*^24^ research.

#### 3.1.3. Functional group analysis

**Fig. 5:**
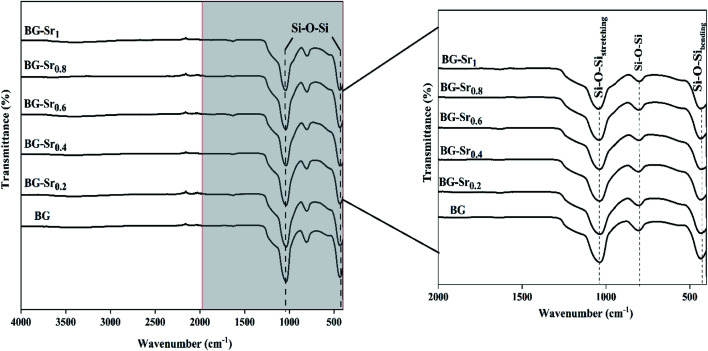
FTIR of different compositions after sintering at 700 °C.

The infrared absorption spectra of the six silicate materials after calcination at 700 °C are shown in (Fig. 5). The spectrum of the synthetic powders showed the most characteristics of bending and stretching vibrations assigned to the silica network existing in bioactive glasses (434, 807, and 1030 cm^−1^). The first peak at 434 cm^−1^ corresponds to the rocking vibration of the Si–O–Si bending mode. The second band was assigned to the symmetric stretching vibration of Si–O. This is related to the stretching vibration of Si–OH bonds, indicating the increase in network connectivity and the formation of more bridging oxygen. The third peak, located at around 1030 cm^−1^, links to the Si–O–Si asymmetric stretching mode, and it is the most pronounced in the spectrum of bioglass. This band represents the silica network of BG-Sr.^51^

Similar bands were observed in previous studies of Leite *et al.* for 55SiO_2_–40CaO–5P_2_O_5_ (mol%) bioactive glass nano-particles^47^ and Zuzana *et al.* for the mesoporous bioactive glass 70 SiO_2_–30CaO (mol%) produced by sol–gel process.^52^ Ta Anh Tuan *et al.* obtained the same results after synthesizing 58SiO_2_–33CaO–9P_2_O_5_ (wt%) using acid-free hydrothermal.^24^ Thus, the XRD and FTIR results approve the success of the hydrothermal method to synthesize an amorphous silicate phase of ternary bioactive glass 63SiO_2_–(37 − *x*)CaO–*x*SrO.

#### 3.1.4. Elemental analysis ICP-AES

Table 4 shows the elementary composition of 63S37C and Sr doped BG using ICP-AES analysis. The experimental compositions of 63S37C BG were revealed to be close to the nominal compositions. Therefore, the latter were first optimized in reference to the compositions already done and their properties. Secondly, according to Hench's explanation,^14^ and in order to not affect the biological properties of the glass, the amount of SiO_2_ and CaO in the binary composition were then selected.

**Table 4:** Molar composition of BG-Sr powders by ICP-AES

Sample acronym	SiO_2_ (mol%)	CaO (mol%)	SrO (mol%)
BG	60.08 ± 0.04	36.02 ± 0.06	0.0000
BG-Sr_0.2_	58.08 ± 0.04	37.77 ± 0.06	0.02 ± 0.04
BG-Sr_0.4_	58.98 ± 0.02	36.88 ± 0.05	0.03 ± 0.05
BG-Sr_0.6_	60.08 ± 0.07	34.47 ± 0.04	0.06 ± 0.03
BG-Sr_0.8_	60.58 ± 0.05	36.76 ± 0.02	0.09 ± 0.04
BG-Sr_1_	61.24 ± 0.03	35.65 ± 0.04	1.06 ± 0.05

Overall, these results suggest the successful hydrothermal synthesis of the glass, with a slight difference. This is due to the heterogeneous distribution of the elements on the amorphous glass matrix. Therefore, XRD analysis (Fig. 4), FTIR (Fig. 5), and ICP-AES (Table 4) demonstrate that hydrothermal synthesis is a suitable method to produce a pure phase of an amorphous binary bioglass 63S37C and the ternary bioglass with different amounts of strontium.

#### 3.1.5. Morphological characteristics

**Fig. 6:**
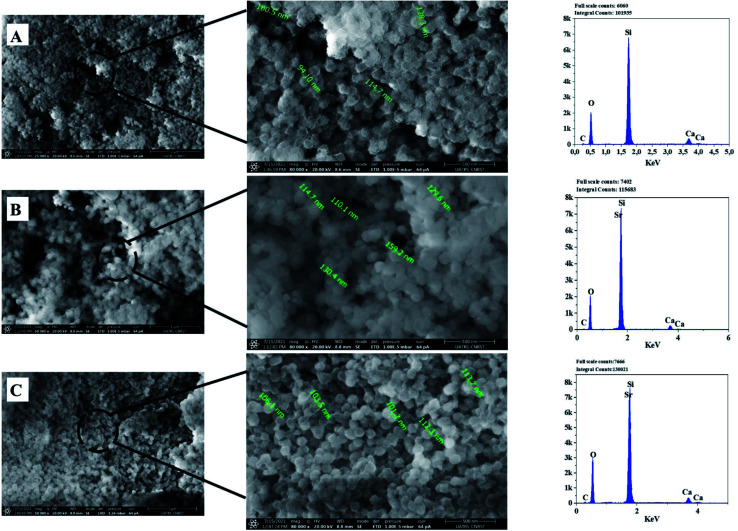
SEM-Energy Dispersive X-ray Spectroscopy (EDS) analyses of the bioactive glass calcined at 700 °C: BG (A), BG-Sr_0.6_ (B), BG-Sr_1_ (C).

Fig. 6 presents the micrographs of 63S37C BG (A) and only two different strontium compositions (BG-Sr_0.6_ (B) and BG-Sr_1_ (C)), to do not encumber the figure since there is a similar tendency with the concentration increase of strontium in bioglass. Even though the particles appear as an aggregation of small particles, irregularly sized, with no significant morphological differences found across the three compositions (BG (A), BG-Sr_0.6_ (B), BG-Sr_1_ (C)), which means that strontium incorporation does not affect the shape of the BG particles.^49,50^ However, strontium ions may modify the particle size, which is explained by one of the conditions for substitution inside a network.^3,42^ Since SiO_2_ is a glass network former, and SrO is a glass network modifier similar to CaO, strontium replaces calcium in bioglass. Moreover, the larger ionic radius of strontium “Sr” (0.112 nm) leads to more open silicate networks than calcium “Ca” (0.099 nm). Thus, the particle size of the synthesized Sr doped bioglasses increases as strontium is enhanced from 0.2 to 1 mol%.^53^ L. Weng *et al.* studied a binary system doped with strontium. They found that the substitution of Ca with Sr increased the nanofibers diameters of BG (from 229 nm to 420 nm). This is due to the higher limiting equivalent ionic conductance of Ca^2+^ compared to Sr^2+^ ions.^54^ Furthermore, with a comparison inspection, the spherical particles, their form, and size become more apparent and denser when the Sr concentration in the chemical composition of BG increases. According to Fig. 6 (BG-Sr_1_ (C)), a weal nanoparticles dispersion was observed. Furthermore, strontium-doped bioactive glass nanoparticles developed in this study show a spherical morphology with a particle size of 90 to 160 nm. The same results were also observed by Ta Anh Tuan *et al.*^24^ These nanospherical particles were formed due to the fusion of BG particles during the hydrothermal process. Moreover, according to the SEM images, the CTAB surfactant assisted-hydrothermal method favors a homogeneous spherical shape and an excellent dispersion of the obtained nanoparticles.

The EDS analysis of the glass nanoparticles shows the evaluation of Ca, Si, and Sr in the particles. The results presented an overlaying peak of Si and Sr, indicating strontium doped 63S37C BG formation. These outcomes agree with the nominal composition, measured by ICP-AES and presented in Table 4. As the literature knows, it is challenging to produce well-dispersed BG particles less than 100 nm since smaller particles are more likely to agglomerate.^23,24,55^ However, in this study and for the first time, spherical nanoparticles (94 nm) have been produced by the hydrothermal method using a CTAB as a surfactant template. Furthermore, all the characterization techniques, DRX, FTIR, ICP, and MEB-EDS, prove that the surfactant-assisted hydrothermal synthesis is suitable for synthesizing spherical nanoparticles bioglasses.

### 3.2. Acellular bioactivity assessment

**Fig. 7:**
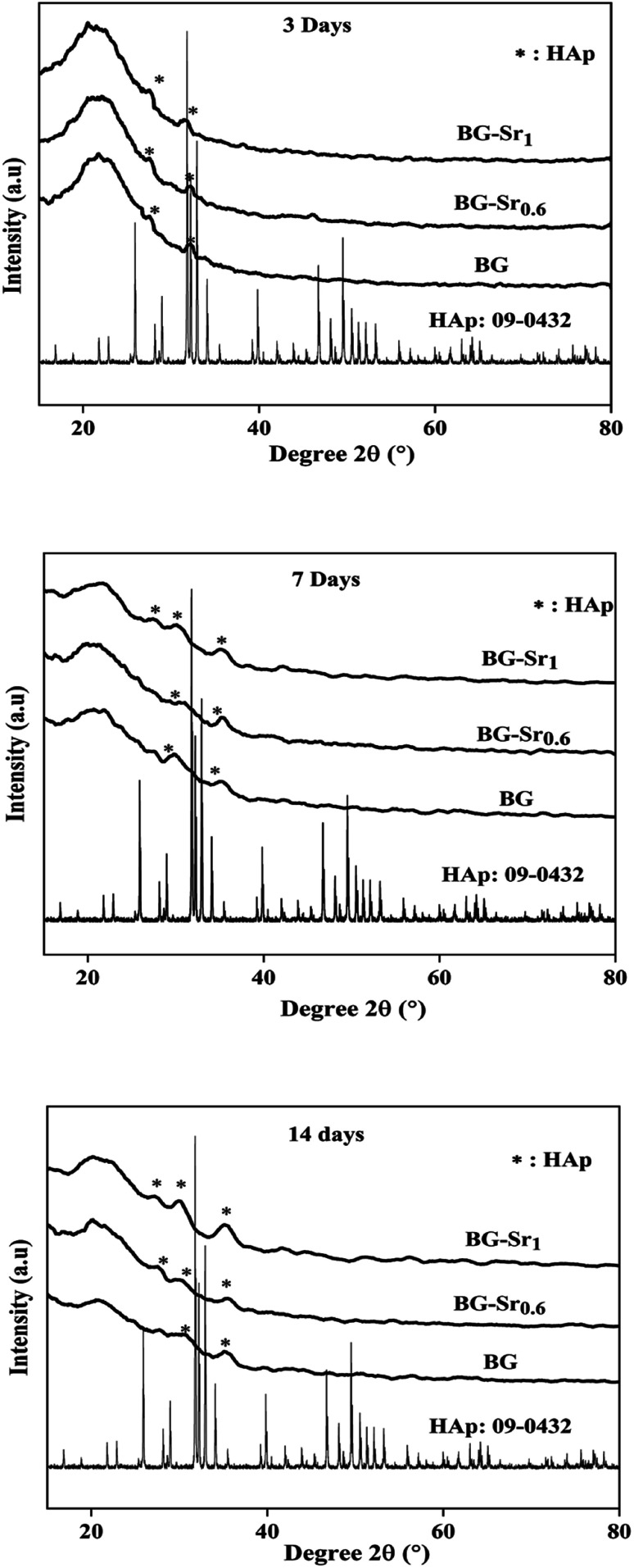
XRD patterns of BG and BG-Sr_*x*_ after immersion in SBF medium for 3, 7 and 14 days.

The assessment of acellular bioactivity in simulated body fluid (SBF) was performed to predict the bone-bonding capability. This could be expressed by forming a new apatite layer on the surface of the new synthesized glasses. Fig. 7 represents the XRD patterns of bioactive glass BG, BG-Sr_0.6,_ and BG-Sr_1_ before and after immersion in SBF solution for 3, 7, and 14 days. The XRD diagrams confirm the formation of a new phase of crystalline hydroxyapatite (HAp) on the surface of nanoparticles BG 63S37C and bioglass doped strontium BG-Sr_0.6_ and BG-Sr_1_, upon only 3 days of immersion in SBF. Furthermore, a slight sharpening of the broadband between 18° and 30° was observed in the XRD pattern after soaking samples for 3 days. This is primarily due to particles size and/or perfection or strain.^56^ After more extended periods (7 to 14 days) of immersion, a slight shift of the peak (between 30° and 32°, and 27° and 29°) toward high theta values is observed. This shift may be attributable to the observed maximum apatite reflection relative to the plan (211). The shifting peak indicated either the difference in composition between the bioactive glass (before soaking) and the forming apatite-like layer and/or the growth of the apatite crystals relative to the period of soaking,^57^ which is the case of samples prepared in this study. Moreover, a new apatite diffraction peak (002) has appeared at 2*θ* = 27°. The intensity of the peak became more pronounced for bioglasses BG-Sr_1_ and BG-Sr_0.6,_ respectively, for a soaking time of 7 to 14 days. This is due to the vigorous growth of hydroxyapatite on the glass surface. The same results were also reported in previous studies.^51,57^ They observed the development hydroxyapatite layer on the surface of bioactive glasses doped with strontium.^57^ They elucidate that Sr^2+^ doping somewhat slows down the production of the apatite because of the inhibitory effects of Sr on the kinetic of mineralization. However, this impact is impertinent to reducing the BG nanoparticles' mineralization capacity.^47^ Likewise, Taherkhani *et al.* discovered new additional firm peaks at 31°, 31.6°, and 39°, respectively, attributed to (301), (002), and (130) planes, and confirmed that the peaks belong to a second crystalline phase of β-Sr_2_SiO_4_.^50,58^ However, the present study displayed a development of a pure apatite phase of glass surfaces as a function of Sr.

These results approve the positive effect of strontium incorporation on the bioactivity of BG, due to its physical and chemical similarity with calcium ions. For the first time, bioactive glass 63SiO_2_–(37 − *x*)CaO–*x*SrO (mol%) was prepared with a primary catalyst by hydrothermal method. It is worth noting that synthetic bioactive glass shows interesting bioactivity by forming an apatite phase after immersion in SBF solution for 14 days and may find potential application as bone substitutes.

**Fig. 8:**
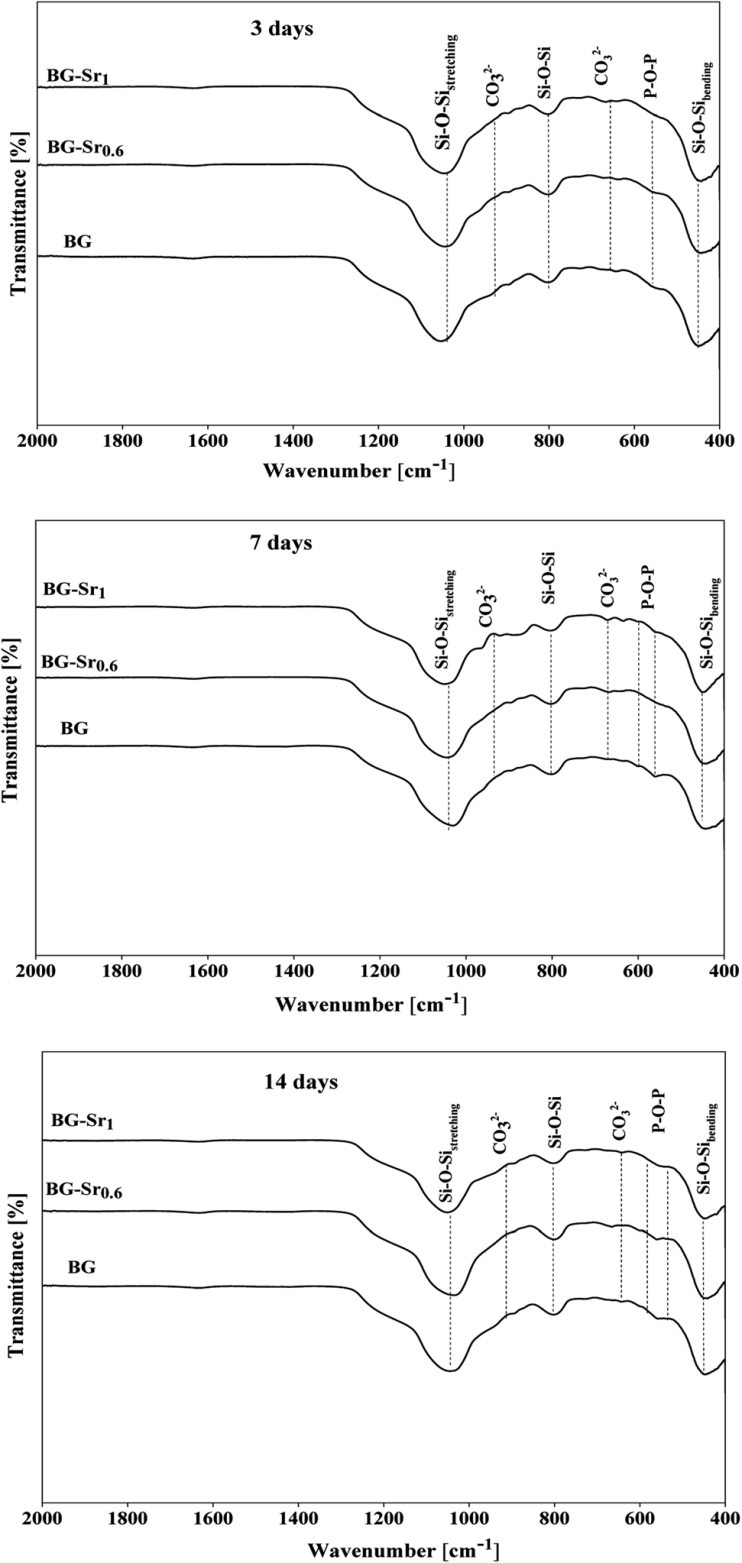
FTIR spectra of BG and BG-Sr_*x*_ after immersion in SBF medium.

These observations were approved by infrared spectroscopy. According to Fig. 8, after only 3 days of immersion in SBF medium, the presence of hydroxyapatite was confirmed by the existence of two bonds at 563 and 630 cm^−1^. These bonds are attributed to the P–O vibrations, which stand to the amorphous Ca–P phase into hydroxyapatite.^39^ These results indicated that the produced BG nanoparticles, newly synthesized by the hydrothermal method using CTAB as a surfactant, induced the growth of hydroxyapatite on their surface.^59^

**Fig. 9:**
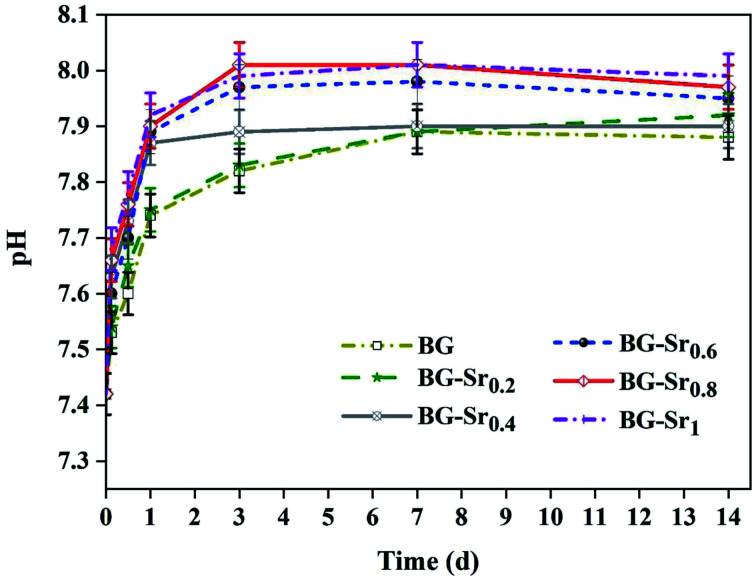
pH variations during the acellular bioactivity tests in SBF medium.

Fig. 9 shows the pH variation of the SBF solution after 3.6 hours and 1, 3, 7, and 14 days of immersion. The plots showed that the pH values increased with the immersion time. This is explained by the exchange of Ca^2+^ ions existing in the silicate compositions with H^+^ ions of the SBF solution. Moreover, according to studies,^13,14,60^ the exchange of Ca^2+^ and H^+^ ions produce Si–OH on the glass surface and enhances the formation of the new apatite layer. Even after 3 days, the pH decreases and stabilizes, meaning that the silica-rich layers are produced, and the cation exchange is prevented. In addition, the highest pH value is related to BG-Sr_1_ due to the high Sr level. Indeed, this result was confirmed by several studies using sol–gel as the synthesis method.^51^

**Fig. 10:**
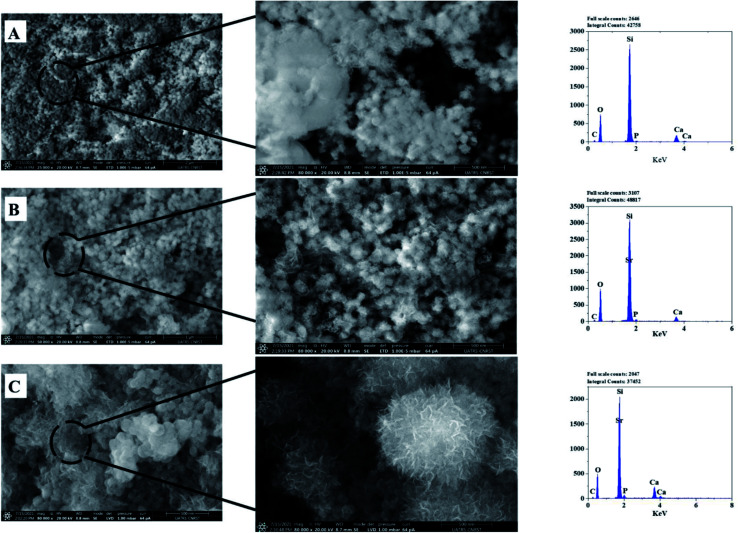
SEM-EDS analysis of bioactive glass after 14 days of immersion.

Fig. 10 depicts the SEM micrographs of the synthesized nanoparticles BG (A), BG-Sr_0.6_ (B), and BG-Sr_1_ (C) after 14 days of immersion in SBF. The samples revealed a dense apatite layer covered the nonspherical particles (94–160 nm). The SEM micrographs showed a typical morphology of the deposited new phase of hydroxyapatite, assembled as needle-like. EDS analysis characterized the evolution of apatite formation. Herein, the nanoparticles presented a gradual increase in the intensity of both calcium and phosphors “P” peaks accompanied by a steady decrease in the silicon “Si” peaks along the 14 days of immersion since the soluble SiO_2_ was lost from the surface of the glass specimens to the SBF solution. These results are in concordance with the observations of L. Hench and co-workers.^14^ Moreover, a second explanation added by A. Moghanian justifies the increase of Si release by increasing the network disorder of doped BG, which is related to the substitution of Ca with Sr, since the latter affects the trending solubility of bioactive glass.^42^ Strontium overlaying peak decreased in the EDS spectrum due to the low concentration among the compositions and because of its exchange with H^+^ in SBF solution, which is expected to increase pH solution as the rise of the immersion period (Fig. 9).

According to FTIR and XRD results after immersion, the deposition of the new apatite layer increase with a longer time (upon 14 days). This is in concordance with SEM micrographs and consistent with previous studies.^57^ For instance, Wei-Hua Lu *et al.* reported that the apatite-like coating becomes very dense with time immersion extended.^13^ The same results were carried out in several studies by Z. Goudarzi, which found that the BG surface becomes covered after 21 days with hydroxyapatite.^51^ Jonathan Lao *et al.* confirmed that the increase of strontium content decreased the dissolution rate of bioactive glass, but the hydroxyapatite layer formation rate is increased.^37^ In addition, the intensity of phosphorus peak after 14 days of soaking in SBF solution is higher in BG-Sr_1_ (C) than in other samples. This indicates a higher tendency to form hydroxyapatite on the glass surface, as proved by XRD results (Fig. 10). This is owing to the Ca/P ratio. According to elementary composition (EDS), the Ca/P ratio obtained in the compositions considered in this study is equal to 1.53, which is near to the one existing in the structure of human bone (1.67). This finding agrees with A. Bakhtiari *et al.*'s study.^61^

### 3.3. Drug loading and release tests

Recently, a great interest has been intensely given to the development of bioactive glass as a drug carrier due to numerous and unique features, such as size, morphology, high surface area, pore-volume, and stable physicochemical characteristics.^62^ In addition, a popular subject of research is targeted drug delivery systems with a variety of drugs, such as ibuprofen, amoxicillin, aspirin, gentamicin, ceftriaxone, and sulbactam sodium.^18,63^ Ibuprofen was selected as a model molecule in this work since it is authentic and frequently used as an anti-inflammatory drug.^64–67^ Furthermore, it has a lipophilic nature and convenient molecular size to be incorporated into the pores of the bioglass nanoparticles.^68^

**Fig. 11:**
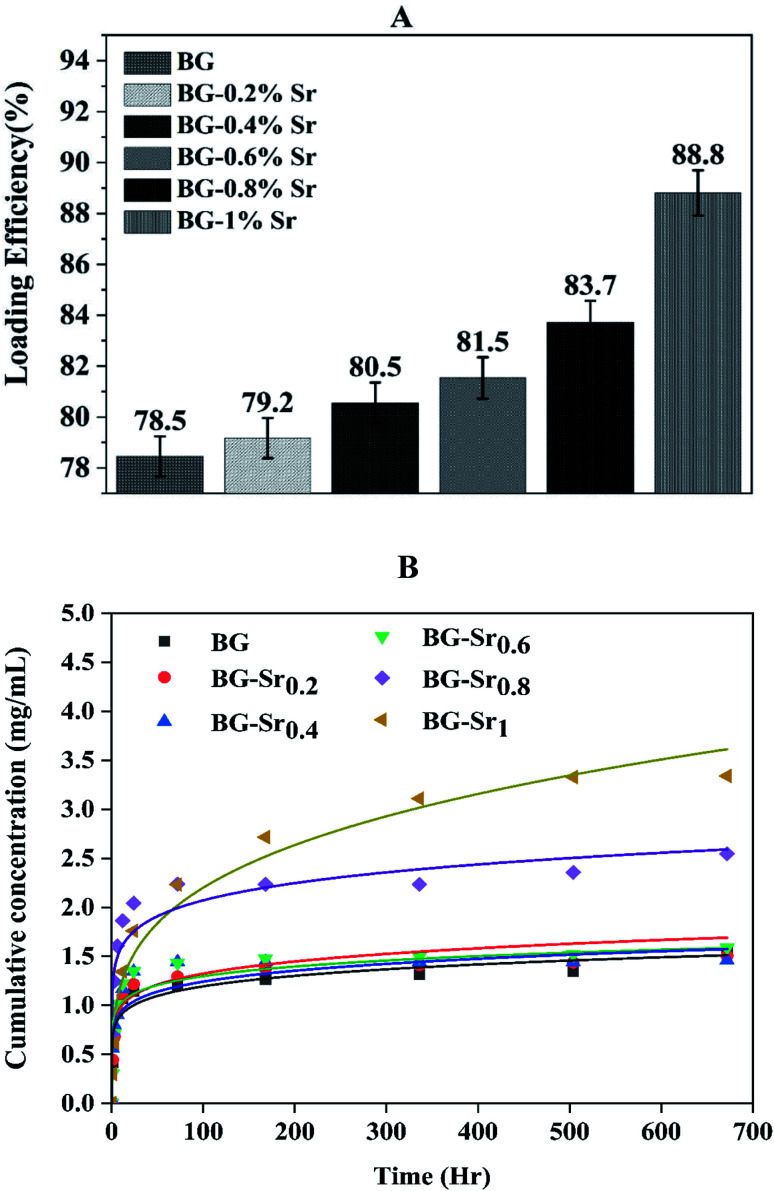
(A) Loading efficiency of ibuprofen with BG and BG-Sr_*x*_ synthesized; (B) release profile of IBU in PBS of the BG and BG-Sr_*x*_ compositions at 37 °C (the solid line is according to KP models' fitting).

Drug loading was achieved using an ethanolic solution of ibuprofen with 2.5 mg ml^−1^. The evolution of IBU concentration inside virgin BG (63S37C) and BG-Sr_*x*_ is presented in Fig. 11 (B) (dot lines). These results indicate that BG-Sr_1_ exhibit the highest absorbance of the IBU drug molecules (88.8%), followed by BG-Sr_0.8_, BG-Sr_0.6_, BG-Sr_0.4_, BG-Sr_0.2_, and BG with 83.7%, 81.5%, 80.5%, 79.2%, and 78.5% respectively. The virgin bioglass 63S37C has the lowest value of IBU loading efficiency. The strontium has then a positive effect on improving the loading efficiency of the ibuprofen molecule, which is undoubtedly related to an increase in the textural properties of bioglasses synthesized by the hydrothermal method, specific surface area. As reported in the literature, a deep study by Vallet-Regi and colleagues emphasized the importance of BG's textural properties to regulate drug loading and release.^12,66^ They explained the different factors controlling the adsorption and delivery phenomena. First, they explain that the decrease in the pore size leads to a decrease in the release rate. Secondly, they reported that the bigger the specific surface area, the greater the amount of drug absorbed. The third factor is related to the pore volume; when it is higher, the number of biomolecules loaded in the mesoporous matrix increases. Finally, they conclude that functionalization also affects molecular absorption and drug loading is linked to surface phenomena.^12,18,66^ By similarity, the results obtained in the present work can be explained by the various factors revealed and confirmed in this last work.

The *in vitro* release of ibuprofen was performed in phosphate-buffered saline PBS (pH = 7.4), with a 1 mg ml^−1^ of BG concentration. This proportion was calculated to make the ratio of the powder to drug equal to unity in each case to simulate the behavior of the material after drug delivery (Fig. 11(B), dot lines). According to this figure, it can be seen that the samples have the same behavior toward IBU release but different cumulative amounts with increasing strontium content in the compositions. During the first period (3 hours), the compositions immediately increase IBU concentration in PBS, finally arriving at a steady state for all samples after 28 days. The release behavior is explained by the drug matrix mixture compression (diffusion constraints) and the surface area and pore volume of bioactive glass, which induce a slow or even incomplete release of ibuprofen.^12,69^

To qualify the mechanism of drug release, various researchers have explained it using various models.^70^ For example, Korsmeyer and Peppas proposed an empirical equation to model Fickian and non-Fickian drug release from different materials (eqn (3)).

3 *M*_g_/*M* = *K*_∞_*t*^*n*^

where:- *M*_g_/*M* is a fraction of drug released at time *t*.- *K* is the rate constant (having units of *t*^*n*^).- *n* value is used to characterize different release mechanisms.

To find out the mechanisms governing the IBU release, the data were fitted in Korsmeyer–Peppas model^69^ (Fig. 11(B), solid lines). According to *n* value, the release process can be predicted: if *n* < 0.5, the mechanism corresponds to Fickian diffusion and if 0.5 < *n* < 1, the mechanism will correspond to non-Fickian transport. For instance, Fickian diffusional release is caused by the drug's typical molecular diffusion due to a potential chemical gradient. However, the non-Fickian transport is due to the matrix's relaxation (erosion) phenomenon. According to the calculated parameters (Table 5), this study's IBU release follows the Fickian diffusion mechanism.

**Table 5:** Parameters of IBU release profile fitting according to Peppas and Korsmeyer model

Model	KP					
Equation	*Kt* ^*n*^					
	BG	BG-Sr_0.2_	BG-Sr_0.4_	BG-Sr_0.6_	BG-Sr_0.8_	BG-Sr_1_
*K*	0.6763 ± 0.06364	0.70122 ± 0.06738	0.79942 ± 0.0791	0.73287 ± 0.10169	1.2065 ± 0.12547	0.6636 ± 0.084
*n*	0.1234 ± 0.01858	0.12412 ± 0.01896	0.10525 ± 0.0199	0.12845 ± 0.02726	0.11737 ± 0.2068	0.26029 ± 0.02248
*R*-square (COD)	0.94696	0.92986	0.90987	0.87144	0.91303	0.97059

In conclusion, ibuprofen release tests for bioglass reveal that the diffusion of ibuprofen molecules does not seem limited, mainly for strontium-doped bioglass due to good porosity and high specific surface area after the insertion of strontium in the silicate matrix. Even though rapid antibiotic delivery is necessary immediately after surgery for effective microbe inhibition, a regulated release is required to avoid high antibiotic concentrations' toxic and systemic effects. This rapid kinetic profile is of great interest for pharmaceutical application, with the aim to enhance the rapid drug delivery of poorly water-soluble drugs, like indomethacin, ibuprofen, and felodipine.^71,72^

## 4. Conclusion

For the first time, a novel 63S37C bioactive glass with a different amount of strontium (0.2–1 mol%) was synthesized by hydrothermal method, with CTAB as surfactant. The hydrothermal process showed that bioglass “63S37C” performed differently from the other synthesis methods (sol–gel and quenched melting). Our findings indicate that hydrothermal is a viable method to produce an amorphous bioactive glass nanoparticle of about 94 nm, with uniform spherical morphology. Indeed, the synthetic bioglass demonstrates notable acellular bioactivity after immersion in SBF medium, by the deposition of a crystallized new apatite layer on the surface of the particles, in a short period of 3 days, especially for BG-Sr_1_ in comparison with the other samples. Furthermore, the inclusion of strontium into the silicate matrix showed excellent properties of loading and delivery of ibuprofen molecule in only 1 hour in PBS solution. This novel bioactive glass may find a potential biomedical application as a bone substitute and drug carrier. The hydrothermal method supplied by a surfactant is an efficient method to synthesize novel bioactive glass spherical nanoparticles with enhanced properties.

## Conflicts of interest

The authors declare that they have no known competing financial interests or personal relationships that could have appeared to influence the work reported in this paper.

## Supplementary Material

## References

[cit1] Hench L. L., Jones J. R. (2015). Front. Bioeng. Biotechnol..

[cit2] Gorustovich A. A., Roether J. A., Boccaccini A. R. (2010). Tissue Eng., Part B.

[cit3] Zheng K., Boccaccini A. R. (2017). Adv. Colloid Interface Sci..

[cit4] Mehrabi T., Mesgar A. S., Mohammadi Z. (2020). ACS Biomater. Sci. Eng..

[cit5] Kaur G., Pandey O. P., Singh K., Homa D., Scott B., Pickrell G. (2014). J. Biomed. Mater. Res., Part A.

[cit6] Plewinski M., Schickle K., Lindner M., Kirsten A., Weber M., Fischer H. (2013). Dent. Mater..

[cit7] Letaief N., Lucas-Girot A., Oudadesse H., Meleard P., Pott T., Jelassi J., Dorbez-Sridi R. (2014). J. Non-Cryst. Solids.

[cit8] Liang Q., Hu Q., Miao G., Yuan B., Chen X. (2015). Mater. Lett..

[cit9] Jones J. R. (2015). Acta Biomater..

[cit10] Martinez A., Izquierdo-Barba I., Vallet-Regi M. (2000). Chem. Mater..

[cit11] Nayak J. P., Bera J. (2010). Appl. Surf. Sci..

[cit12] Vallet-Regi M., Rámila A., del Real R. P., Pérez-Pariente J. (2001). Chem. Mater..

[cit13] Lu W. H., Li K. D., Lu C. H., Teoh L. G., Wu W. H., Shen Y. C. (2013). Mater. Trans..

[cit14] Hench L. L. (2006). J. Mater. Sci.: Mater. Med..

[cit15] Lehman L. F. C., Noronha M. S., Diniz I. M. A., Costa e Silva R. M. F., Andrade Â. L., Sousa Lima L. F., Alcântara C. E. P., Domingues R., Ferreira A. J., Silva T. A., Mesquita R. A. (2019). J. Tissue Eng. Regener. Med..

[cit16] Li R., Clark A. E., Hench L. L. (1991). J. Appl. Biomater..

[cit17] Hench L. L., Polak J. M. (2002). Science.

[cit18] Tabia Z., El Mabrouk K., Bricha M., Nouneh K. (2019). RSC Adv..

[cit19] Yan X., Yu C., Zhou X., Tang J., Zhao D. (2004). Angew. Chem., Int. Ed..

[cit20] Hu Q., Li Y., Zhao N., Ning C., Chen X. (2014). Mater. Lett..

[cit21] Zhu Y., Kaskel S. (2009). Microporous Mesoporous Mater..

[cit22] Gan Y. X., Jayatissa A. H., Yu Z., Chen X., Li M. (2020). J. Nanomater..

[cit23] Hoa B. T., Hoa H. T. T., Tien N. A., Khang N. H. D., Guseva E. V., Tuan T. A., Vuong B. X. (2020). Mater. Lett..

[cit24] Tuan T. A., Guseva E. V., Phuc L. H., Hien N. Q., Long N. V., Vuong B. X. (2020). Proceedings.

[cit25] Hench L. L., Wheeler D. L., Greenspan D. C. (1998). J. Sol-Gel Sci. Technol..

[cit26] Jones J. R., Sepulveda P., Hench L. L. (2001). J. Biomed. Mater. Res..

[cit27] Bricha M., Nivedha V., Ramadas M., El Mabrouk K., Ballamurugan A. M. (2018). Trends Biomater. Artif. Organs.

[cit28] Tabia Z., El Mabrouk K., Bricha M., Nouneh K. (2019). RSC Adv..

[cit29] Akhtach S., Tabia Z., El Mabrouk K., Bricha M., Belkhou R. (2021). Ceram. Int..

[cit30] Philippart A., Gómez-Cerezo N., Arcos D., Salinas A. J., Boccardi E., Vallet-Regi M., Boccaccini A. R. (2017). J. Non-Cryst. Solids.

[cit31] Bano S., Akhtar M., Yasir M., Maqbool M. S., Niaz A., Wadood A., Rehman M. A. U. (2021). Gels.

[cit32] Bonnelye E., Chabadel A., Saltel F., Jurdic P. (2008). Bone.

[cit33] Gentleman E., Fredholm Y. C., Jell G., Lotfibakhshaiesh N., O'Donnell M. D., Hill R. G., Stevens M. M. (2010). Biomaterials.

[cit34] Isaac J., Nohra J., Lao J., Jallot E., Nedelec J. M., Berdal A., Sautier J. M. (2011). Eur. Cells Mater..

[cit35] Wu C., Chang J. (2014). J. Controlled Release.

[cit36] Fredholm Y. C., Karpukhina N., Brauer D. S., Jones J. R., Law R. V., Hill R. G. (2012). J. R. Soc., Interface.

[cit37] Lao J., Nedelec J. M., Jallot E. (2009). J. Mater. Chem..

[cit38] Slowing I. I., Trewyn B. G., Giri S., Lin V. S. Y. (2007). Adv. Funct. Mater..

[cit39] Kokubo T., Takadama H. (2006). Biomaterials.

[cit40] Aghaei H., Nourbakhsh A. A., Karbasi S., JavadKalbasi R., Rafienia M., Nourbakhsh N., Bonakdar S., Mackenzie K. J. D. (2014). Ceram. Int..

[cit41] Oliveira I. R., Barbosa A. M., Santos K. W., Lança M. C., Lima M. M. R. A., Vieira T., Silva J. C., Borges J. P. (2022). Ceram. Int..

[cit42] Moghanian A., Firoozi S., Tahriri M. (2017). Ceram. Int..

[cit43] Amudha S., Ramya J. R., Arul K. T., Deepika A., Sathiamurthi P., Mohana B., Asokan K., Dong C. L., Kalkura S. N. (2020). Composites, Part B.

[cit44] Edén M., Sundberg P., Stålhandske C. (2011). J. Non-Cryst. Solids.

[cit45] Rocton N., Oudadesse H., Lefeuvre B. (2018). Thermochim. Acta.

[cit46] Bellucci D., Sola A., Salvatori R., Anesi A., Chiarini L., Cannillo V. (2017). Mater. Sci. Eng., C.

[cit47] Leite Á. J., Gonçalves A. I., Rodrigues M. T., Gomes M. E., Mano J. F. (2018). ACS Appl. Mater. Interfaces.

[cit48] Galliano P. G., López J. M. P., Varetti E. L., Sobrados I., Sanz J. (1994). Mater. Res. Bull..

[cit49] Massera J., Hupa L. (2014). J. Mater. Sci.: Mater. Med..

[cit50] Taherkhani S., Moztarzadeh F. (2016). J. Sol-Gel Sci. Technol..

[cit51] Goudarzi Z., Ijadi A., Bakhriari A., Eskandarinezhad S., Azizabadi N., Asgari Jazi M. (2020). J. Compos. Compd..

[cit52] Neščáková Z., Zheng K., Liverani L., Nawaz Q., Galusková D., Kaňková H., Michálek M., Galusek D., Boccaccini A. R. (2019). Bioact. Mater..

[cit53] Fredholm Y. C., Karpukhina N., Law R. V., Hill R. G. (2010). J. Non-Cryst. Solids.

[cit54] Weng L., Boda S. K., Teusink M. J., Shuler F. D., Li X., Xie J. (2017). ACS Appl. Mater. Interfaces.

[cit55] Stark W. J., Pratsinis S. E. (2002). Powder Technol..

[cit56] Vallet-Regí M., Romero A. M., Ragel C. V., LeGeros R. Z. (1999). J. Biomed. Mater. Res..

[cit57] Leite Á. J., Caridade S. G., Mano J. F. (2016). J. Non-Cryst. Solids.

[cit58] Dai L. L., Nudelman F., Chu C. H., Lo E. C. M., Mei M. L. (2021). J. Dent..

[cit59] Kim J. H., Kim S. H., Kim H. K., Akaike T., Kim S. C. (2002). J. Biomed. Mater. Res..

[cit60] Beltrán A. M., Begines B., Alcudia A., Rodríguez-Ortiz J. A., Torres Y. (2020). ACS Appl. Mater. Interfaces.

[cit61] Bakhtiari A., Cheshmi A., Naeimi M., Mohammadi Fathabad S., Aliasghari M., Modarresi Chahardehi A., Hassani S., Elhami V. (2020). J. Compos. Compd..

[cit62] He Q., Shi J. (2011). J. Mater. Chem..

[cit63] Anand A., Das P., Nandi S. K., Kundu B. (2020). Ceram. Int..

[cit64] Ghaebi Panah N., Alizadeh P., Eftekhari Yekta B., Motakef-Kazemi N. (2016). Ceram. Int..

[cit65] Muñoz B., Rámila A., Pérez-Pariente J., Díaz I., Vallet-Regí M. (2003). Chem. Mater..

[cit66] Manzano M., Colilla M., Vallet-Reg M. (2009). Expert Opin. Drug Delivery.

[cit67] Araújo M., Viveiros R., Philippart A., Miola M., Doumett S., Baldi G., Perez J., Boccaccini A. R., Aguiar-Ricardo A., Verné E. (2017). Mater. Sci. Eng., C.

[cit68] Wang X., Liu P., Tian Y. (2011). Microporous Mesoporous Mater..

[cit69] Taghzouti O. K., El Mabrouk K., Bricha M., Nouneh K. (2019). SN Appl. Sci..

[cit70] Paarakh M. P., Jose P. A. N. I., Setty C. M., Peter G. V. (2019). Int. J. Pharm. Res. Technol..

[cit71] Boyd B. J., Bergström C. A. S., Vinarov Z., Kuentz M., Brouwers J., Augustijns P., Brandl M., Bernkop-Schnürch A., Shrestha N., Préat V., Müllertz A., Bauer-Brandl A., Jannin V. (2019). Eur. J. Pharm. Sci..

[cit72] Charnay C., Bégu S., Tourné-Péteilh C., Nicole L., Lerner D. A., Devoisselle J. M. (2004). Eur. J. Pharm. Biopharm..

